# Heterologous Combination of ChAdOx1 and MVA Vectors Expressing Protein NS1 as Vaccination Strategy to Induce Durable and Cross-Protective CD8+ T Cell Immunity to Bluetongue Virus

**DOI:** 10.3390/vaccines8030346

**Published:** 2020-06-29

**Authors:** Sergio Utrilla-Trigo, Luis Jiménez-Cabello, Ruymán Alonso-Ravelo, Eva Calvo-Pinilla, Alejandro Marín-López, Sandra Moreno, Gema Lorenzo, Julio Benavides, Sarah Gilbert, Aitor Nogales, Javier Ortego

**Affiliations:** 1 Centro de Investigación en Sanidad Animal (CISA), Instituto Nacional de Investigación y Tecnología Agraria y Alimentaria (INIA), Valdeolmos, 28130 Madrid, Spain; sergio.utrilla@ucm.es (S.U.-T.); luisfjim@ucm.es (L.J.-C.); ralonso0410@gmail.com (R.A.-R.); calvo.eva@inia.es (E.C.-P.); alejandro.marinlopez@yale.edu (A.M.-L.); sandramorenofdez@gmail.com (S.M.); lorenzo.gema@inia.es (G.L.); nogales.aitor@inia.es (A.N.); 2 Instituto de Ganadería de Montaña (CSIC-Universidad de León), 24346 León, Spain; julio.benavides@csic.es; 3 The Jenner Institute, University of Oxford, Oxford OX3 7DQ, UK; sarah.gilbert@ndm.ox.ac.uk

**Keywords:** bluetongue virus (BTV), modified vaccinia Ankara (MVA), chimpanzee adenovirus (ChAdOx1), vaccine, cellular response, protein NS1

## Abstract

The sequence of non-structural protein NS1 of bluetongue virus (BTV), which contains immunodominant CD8+ T cell epitopes, is highly conserved among BTV serotypes, and has therefore become a major tool in the development of a universal BTV vaccine. In this work, we have engineered multiserotype BTV vaccine candidates based on recombinant chimpanzee adenovirus (ChAdOx1) and modified vaccinia virus Ankara (MVA) vectors expressing the NS1 protein of BTV-4 or its truncated form NS1-Nt. A single dose of ChAdOx1-NS1 or ChAdOx1-NS1-Nt induced a moderate CD8+ T cell response and protected IFNAR(-/-) mice against a lethal dose of BTV-4/MOR09, a reassortant strain between BTV-1 and BTV-4, although the animals showed low viremia after infection. Furthermore, IFNAR(-/-) mice immunized with a single dose of ChAdOx1-NS1 were protected after challenge with a lethal dose of BTV-8 in absence of viremia nor clinical signs. Additionally, the heterologous prime-boost ChAdOx1/MVA expressing NS1 or NS1-Nt elicited a robust NS1 specific CD8+ T cell response and protected the animals against BTV-4/MOR09 even 16 weeks after immunization, with undetectable levels of viremia at any time after challenge. Subsequently, the best immunization strategy based on ChAdOx1/MVA-NS1 was assayed in sheep. Non-immunized animals presented fever and viremia levels up to 10^4^ PFU/mL after infection. In contrast, although viremia was detected in immunized sheep, the level of virus in blood was 100 times lower than in non-immunized animals in absence of clinical signs.

## 1. Introduction

Bluetongue is a vector-borne viral disease that affects both domestic and wild ruminants and is characterized by vascular injury resulting in tissue necrosis, hemorrhage and edema, among other lesions [1]. This disease is caused by bluetongue virus (BTV), one of the most important livestock pathogens worldwide [2]. This non-enveloped virus that belongs to the genus *Orbivirus* within the family *Reoviridae* is characterized by its icosahedral layered capsid. It possesses a double-stranded RNA genome divided into ten segments which encode for seven structural proteins named VP1-VP7 as well as six non-structural ones (NS1, NS2, NS3/3A, NS4 and NS5). Historically, the virus has been prevalent in tropical and subtropical regions located between 35 °S and 45 °N, which coincided with the distribution of midges from the genus *Culicoides*, the main vector involved in BTV transmission. However, several outbreaks in regions located further north have been reported, mainly facilitated by the recent spread of insect vectors [3,4,5,6]. To date, 29 serotypes of BTV have been identified [7], with two more putative serotypes and several other variants being further described. At this time, the described serotypes of BTV are classified as typical, ranging from 1 to 24, recognized by cross-neutralization assays and confirmed by phylogenetic analysis [1], and atypical (25–27) [2,3,4,7], found exclusively in small ruminants, non-pathogenic and spread by direct contact transmission [8,9,10,11]. Additionally, serotypes BTV-28 and BTV-29, which are closely related to other typical BTV serotypes, have recently been described, and two others are being studied [4,12,13].

Since 1998, at least 8 different serotypes (1, 2, 4, 6, 8, 9, 11 and 16) have been introduced in the European continent [8]. Moreover, the constant arrival of new serotypes, including the introduction of novel serotypes in endemic regions, highlights the importance of a multiserotype vaccine to control the disease, reducing the suffering of animals and allowing international trade without restrictions thereupon.

Vaccination is the most effective control measure to reduce and eradicate insect-borne diseases [11]. Traditionally, vaccines against BTV have been based on attenuated or inactivated viruses. However, these vaccines are serotype-specific and they are associated with clinical signs and a short viremia that allows transmission as well as risk of reassortment events of genomic segments from different BTV serotypes in the case of live attenuated vaccines [9,13]. Additionally, these vaccines target mostly protein VP2, which represents the main inductor of neutralizing antibodies but has a highly variable antigen sequence among serotypes (up to 72.9% aa divergence) [12]. These conventional vaccines have been useful to control or limit BTV expansion so far, but they are not suitable for cross-protection between serotypes and do not permit the differentiation between infected and vaccinated animals (DIVA strategy) [14,15]. Non-structural proteins have been found to play a key role in several aspects of the infection such as virulence and replication and have therefore been studied as targets for antiviral therapies [16,17]. Among them, NS1 is the most abundantly viral protein produced in infected cells. This protein contains epitopes related with T-cell-mediated responses which may play a major role in protection against BTV [18,19]. Further, its amino acid sequence is highly conserved among serotypes [20,21] which makes it a clear candidate for the development of universal multiserotype vaccines.

Modified vaccinia virus Ankara (MVA) has been extensively used as a viral vaccine vector to induce strong T-cell mediated responses directed towards intracellular pathogens [22]. Recent studies carried out in our laboratory showed that homologous prime-boost immunization of IFNAR(-/-) mice with a recombinant MVA expressing NS1 or its truncated form NS1-Nt protects animals against a lethal challenge with several BTV serotypes, protection that is largely dependent on CD8+ T cell responses [14]. Although the homologous prime-boost with MVA-NS1 or MVA-NS1-Nt showed efficacy in protection against BTV, a heterologous prime-boost combining MVA with a different viral vector could avoid a possible anti-vector immunity and improve the vaccination strategy.

Adenoviruses have also been widely used as vaccine vectors against numerous infectious diseases ranging from malaria to HIV-1 due to the strong innate and adaptive immune responses that they elicit in mammalian hosts [23,24]. Simian adenoviruses, which include replication-defective chimpanzee adenoviruses (ChAd), have lately experimented a boom in their usage as vaccine vectors since they not only are safe and induce strong cellular and humoral immunity but they also lack problems related with pre-existing immunity that has somewhat limited the efficacy of human adenoviruses [25,26]. Vaccine vectors based on recombinant ChAdOx1 have been tested with promising results in many animal models for numerous infectious diseases such as Ebola [25,27], influenza A [28], chikungunya [29] or MERS-CoV [30,31,32], and they are also being evaluated as a vaccine platform against the current pandemic caused by SARS-CoV-2 [33,34]. Furthermore, immunization with ChAdOx1-GnGc vaccine, encoding Rift Valley fever virus (RVFV) envelope glycoproteins, provides solid protection against RVFV challenge in the most susceptible natural target species of the virus, which are sheep, goats and cattle [35], same natural hosts of BTV. The use of replication-defective viral vectors ChAdOx1 and MVA for antigen delivery primes strong CD8+ and CD4+ T cell responses and their use in heterologous prime-boost immunization strategies expressing the same antigens elicits long-lasting cellular immunity [25,27,28,36].

In this work, we have analyzed the protective capacity of protein NS1 or its truncated version NS1-Nt from BTV-4 when they are delivered by the viral vector ChAdOx1 alone or in combination with MVA. Moreover, we evaluated the cellular immune response elicited by these vaccine candidates and their protective efficacy against the virulent reassortant strain BTV-4M in the short and long term in IFNAR(-/-) mice. Furthermore, the best immunization strategy observed in mice was also assayed in sheep, a BTV natural host.

## 2. Materials and Methods

### 2.1. Cells and Viruses

**Figure 1:**
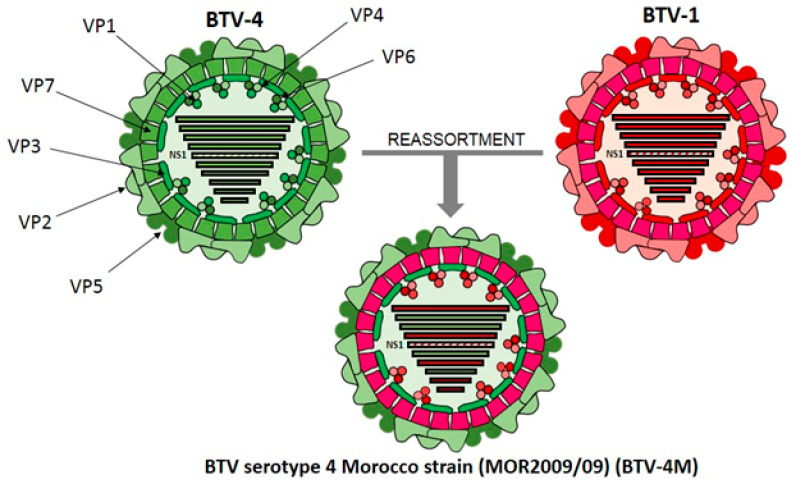
Schematic representation of BTV-4 Morocco, a bluetongue virus (BTV) strain generated as a consequence of a reassortment event between BTV-4 and BTV-1 serotypes. Three concentric protein layers constituted by VP2 and VP5 (outer capsid), VP7 (intermediate layer) and VP3 (subcore), are depicted. Structural proteins VP1, VP4 and VP6 are located inside the inner capsid constituting the RNA polymerase complex. Double-strand RNA segments 1 (VP1), 4 (VP4), 5 (NS1), 7 (VP7), 9 (VP6 and NS4) and 10 (NS3/NS3A and NS5), deriving from BTV-1, are represented in red. Double-strand RNA segments 2 (VP2), 3 (VP3), 6 (VP5) and 8 (NS2), deriving from BTV-4, are colored green. BTV dsRNA segment 5, encoding for NS1, is striped.

Chicken embryo fibroblasts (DF-1) (ATCC, Cat. No. CRL-12203), human embryo kidney cells (HEK-293) (ATCC, Cat. No. CRL-11268G-1), and green monkey kidney cells (Vero) (ATCC, Cat. No. CCL-81) were grown in Dulbecco’s Modified Eagle’s medium (DMEM) (Biowest, Nuaillé, France) supplemented with 2 mM glutamine (Gibco, Waltham, MA, USA ) and 10% fetal calf serum (FCS) (Gibco, Waltham, MA, USA). BTV serotype 4 Morocco strain (MOR2009/09) (BTV-4M) and BTV serotype 8 (BEL/2006) (BTV-8) were used in the experiments. BTV-4M is a reassortant strain between BTV-1 (segments 1, 4, 5, 7, 9, 10) and BTV-4 (segments 2, 3, 6, 8) isolated from sheep blood in KC insect cells [37,38] (Figure 1). BTV-4M, BTV-8, and MVA virus stocks and titrations were performed as previously described [39].

### 2.2. Generation of Recombinant Candidate Vaccine Vectors

The generation of MVA-NS1 and MVA-NS1-Nt have been previously described [14,40,41]. To generate 1990_pENTR4-LPTOS (p1990) adenovirus entry vector containing NS1 or NS1-Nt genes from BTV-4 (SPA2004/02), plasmids pcDNA3-NS1 and pcDNA3-NS1-Nt [14,40,41] were digested with *EcoRI* and *NotI* and the DNA inserts were cloned in the plasmid p1990 previously digested with the same restriction enzymes. Subsequently, plasmids p1990-NS1 and p1990-NS1-Nt were sequenced and sent to “The Jenner Institute viral vector core facility” in the University of Oxford for the ChAdOx1-NS1 and ChAdOx1-NS1-Nt generation and purification.

### 2.3. Indirect Immunofluorescence Microscopy

DF-1 and HEK-293 cells were grown in glass coverslips and infected with MVA-NS1 and MVA-NS1-Nt, or ChAdOx1-NS1 and ChAdOx1-NS1-Nt at a multiplicity of infection (MOI) of 1, respectively. 18 h after infection, the cells were fixed for 15 min with 4% paraformaldehyde and permeabilized with 0.5% Triton-X100 in PBS. Fixed cells were blocked with 20% FBS in PBS (20% blocking solution) for 60 min at room temperature (RT). Cells were then incubated overnight at 4 °C with a mouse hyperimmune serum against BTV-16 (obtained from an in vivo infection [14]) diluted in PBS—20% FBS. After three serial washing steps with PBS, cells were incubated with Alexa Fluor 488 or 594 goat conjugated anti-mouse IgGs (Invitrogen, German Town, MD, USA) for 30 min at RT. Coverslips were washed five times with PBS and once with PBS-DAPI (1:10,000), mounted on glass slides and visualized in a Zeiss Axio fluorescence microscope (Zeiss, Oberkochen, Germany). Images were further processed using the Zen Zeiss software (Zeiss, Oberkochen, Germany).

### 2.4. Mice and Sheep

Type I interferon receptor defective mice (IFNAR (-/-)) on a 129 Sv/Ev background [42], and sheep (Spanish “Churra” sheep breed), were used for the studies. Mice and sheep were housed under pathogen-free conditions and allowed to acclimatize to the biosafety level 3 (BSL3) animal facilities at the Animal Health Research Center (INIA-CISA), Madrid, before use. All mice used were matched for age (8 weeks). Animal experimental protocols were approved by the Ethical Review Committee at the INIA-CISA and Comunidad de Madrid (Permit number: PROEX 172/17) in strict accordance with EU guidelines 2010/63/UE about protection of animals used for experimentation and other scientific purposes and Spanish Animal Welfare Act 32/2007. All efforts were made to minimize suffering.

### 2.5. Immunization of Mice and Challenge

Three different immunization strategies were used in this study: two groups of IFNAR(-/-) mice (*n* = 5) were immunized with a single dose of 10^8^ IU per mouse of either ChAdOx1-NS1 or ChAdOx1-NS1-Nt, two other groups of IFNAR(-/-) mice (*n* = 5) were immunized with a single dose of 10^7^ PFU per mouse of MVA-NS1 or MVA-NS1-Nt, and two additional groups of IFNAR(-/-) mice (*n* = 5) were immunized following a heterologous prime-boost regimen consisting of an initial dose of 10^8^ IU per mouse of ChAdOx1-NS1 or ChAdOx1-NS1-Nt (prime) followed by a second dose of 10^7^ PFU per mouse of MVA-NS1 or MVA-NS1-Nt (boost), respectively, administered 28 days apart. The recombinant MVA vectors were inoculated intraperitoneally whereas immunization with the corresponding ChAdOx1 was carried out via intramuscular injection on the right leg of each mouse. For each study, one group of mice (*n* = 5) was left untreated (non-immunized). Animals were challenged with a lethal dose (10 PFU) of BTV-4M or BTV-8 at 30 days (ChAdOx1 prime-only) or 15 days (MVA prime-only and ChAdOx1/MVA prime-boost) after last immunization. Mice were bled after virus challenge at 3, 5, 7, 10 and 17 days post infection (d.p.i.) for the analysis of viremia. For the experiment of long-lasting protection mice were infected with 10 PFU of BTV-4M at day 154 and bled after challenged at the same time points indicated above to analyze viremia (Figure S1).

Animals were evaluated and scored for individual clinical signs. Rough hair (absent = 0, slightly = 1, markedly = 2), activity (normal = 0, slightly reduced = 1, reduced = 2, severely reduced = 3), eye swelling (absent = 0, slightly = 1, moderate = 2, severe = 3) and temperature (normal = 0, hypothermia = 3). The final score was the addition of each individual score. The minimum score was 0 for healthy and 1–11 depending upon the severity. Animals that reached 8 points of score were euthanized. Each score represents the value of a single animal.

### 2.6. Immunization of Sheep and Challenge

Six naïve healthy sheep (Spanish “Churra” sheep breed), aged four months, were acclimated for seven days at the BSL-3 animal facility of the Animal Health Research Center (INIA-CISA) before starting the experiment. All sheep were negative to BTV by ELISA. Three animals were subcutaneously inoculated with 10^9^ IU of ChAdOx1-NS1 and 10^8^ PFU of MVA-NS1 at days 0 and 30 of the experiment. Five weeks post-booster (w.p.b.), pre-challenge blood samples were collected from all animals and immunized and non-immunized sheep were challenged subcutaneously with 10^5^ PFU of BTV-4M. After challenge, all sheep were monitored daily for clinical signs and rectal temperature. Blood samples for virological analyses were collected at 0, 2, 4, 6, 8 and 11 days post-infection (d.p.i.). The fever threshold was set to ≥40.5 °C based on the mean plus three standard deviations of the rectal temperatures recorded in the six sheep for one week before challenge (Supplementary Materials Figure S2).

### 2.7. Viremia Analysis by RT-qPCR

Blood samples were collected at 3, 5, 7, 10, and 17 d.p.i from mice and at 0, 2, 4, 6, 8 and 11 d.p.i from sheep. RNA was extracted from 50 µL of blood using Trizol Reagent (Sigma Aldrich, St. Louis, MO, USA) following the protocol established by the manufacturer. Viremia was analyzed in duplicate by real-time RT-qPCR specific for BTV segment 5 (encoding for NS1). The real-time RT-qPCR specific for BTV segment 5 was performed using primers and probe described by Toussaint et al. [43]. Briefly, RT-PCR was carried out with the AgPath-ID™ One-Step RT-PCR Reagents (Thermo Fisher Scientific) using 400 nM of each primer (GGCAACYACCAAACATGGA and AAAGTYCTCGTGGCATTWGC) (Sigma Aldrich, St. Louis, MO, USA), 200 nM of the Taqman probe conjugated to FAM at the 5-end and to TAMRA at the 3-end (FAM-CYCCACTGATRTTGTATTTTCTCAA-TAMRA) (Sigma Aldrich, St. Louis, MO, USA) and 2 µL of RNA extracted from 50 µl of blood. Cycling conditions were as follows: 1 cycle at 48 °C for 25 min, 1 cycle of 95 °C for 15 min, 45 cycles with 15 s at 95 °C and 1 min at 61 °C. Ct values ≤ 38 were considered positive. Mouse and sheep blood containing different concentrations of virus (10 PFU/mL to 10^5^ PFU/mL) were titrated and used as standards [14].

### 2.8. Detection of Antibodies against NS1 by ELISA

MaxiSorp plates (Nunc, Rochester, NY, USA) were coated with NS1 purified baculovirus expressed proteins (164 ng per well) and incubated overnight at 4 °C. Plates were saturated with blocking buffer (PBS 0.05% Tween 20 and 5% skim milk). The animal sera diluted in blocking buffer were added and incubated for 1 h at 37 °C. After three washes in PBS—0.05% Tween 20, plates were incubated for 1 h at 37 °C with an anti-mouse-HRP secondary antibody (BioRad, Portland, ME, USA) at a 1/2000 dilution in blocking buffer. Finally, after three washes in PBS—0.05% Tween 20, the reaction was developed with 50 µL of substrate solution 3,3′, 5,5′–tetramethylbencidine liquidsupersensitive (TMB) (Sigma Aldrich, St. Louis, MO, USA) and stopped by adding 50 µL of 3 N H_2_SO_4_ (Merck KGaA, Darmstadt, Germany). Results were expressed as optical densities (ODs) measured at 450 nm.

### 2.9. Plaque Reduction Neutralization Test (PRNT)

Two fold dilutions (from 1:5) of heat inactivated sera (56 °C for 30 min) were incubated with 100 PFU of BTV-4 for 1 h at 37 °C. Then, samples were inoculated into 12-well plates containing semi-confluent monolayers of Vero cells. Following incubation for 1 h an agar overlay (DMEM, 10% FBS, 0.4% Noble Agar (Becton Dickinson, Sparks, MD, USA)) was added and plates incubated for 5 days at 37 °C in 5% CO_2_. Plaques were fixed with 10% formaldehyde and visualized with 2% crystal violet PBS. PRNT50 titer was calculated as the reciprocal (log 10) of the highest dilution of serum that neutralized 50% of the control virus input. The cut-off is 0.69, log of the reciprocal of the first dilution 1:5.

### 2.10. Flow Cytometric Analysis

Groups of IFNAR(-/-) mice (*n* = 4) were immunized with a single dose of ChAdOx1-NS1 or ChAdOx1-NS1-Nt or following a homologous prime-boost regimen with ChAdOx1-NS1 and MVA-NS1 or ChAdOx1-NS1-Nt and MVA-NS1-Nt. Mice were euthanized 15 days post-boost (prime-boost strategy) or 30 days post-immunization (single dose) and their spleens were harvested for analysis by intracellular cytokine staining (ICCS). A total of 10^6^ splenocytes were stimulated with 10 µg/mL of either NS1-152 peptide (9-mer peptide GQIVNPTFI) [44] or a peptide from the VP2 protein (irrelevant peptide) for 5 h in RPMI 1640 supplemented with 10% FCS and containing brefeldin A (2 µg/mL) to enhance IFN-γ accumulation in the responding cells. CD107a/LAMP-1-FITC antibody (Miltenyi Biotec, Bergisch Gladbach, Germany) was also added as indirect marker of cytotoxicity. After stimulation, cells were washed, stained for the surface markers, fixed, permeabilized, and stained intracellularly using the corresponding fluorochromes. The fluorochrome conjugated antibodies CD8-PerCP-Vio700 and IFN-γ–PE (Miltenyi Biotec, Bergisch Gladbach, Germany) were used for the analysis. Data were acquired by FACS analysis on a FACSCalibur platform (Becton Dickinson, Franklin Lakes, NJ, USA). Analyses of the data were performed using FlowJo software version ×0.7 (Tree Star, Ashland, OR, USA). The number of lymphocyte-gated events was 5 × 10^5^. Lymphocytes were initially gated on the basis of their forward and side scatter properties. Then, CD8+ lymphocytes expressing IFN-γ or CD107a were selected for the analysis.

### 2.11. Cytokine Analysis

Splenocytes from immunized and non-immunized mice were stimulated for 72 h with 10 µg/mL of NS1-152 peptide or left untreated in RPMI 1640 supplemented with 10% FCS. Supernatant cytokine levels were analyzed using a multiplex fluorescent bead immunoassay for quantitative detection of mouse cytokines (Millipore’s MILLIPLEX Mouse Cytokine kit, Burlington, MA, USA). Samples were analyzed with a MAGPIX system (Luminex Corporation, Austin, TX, USA). Values of non-stimulated samples were subtracted from values of stimulated samples.

### 2.12. Statistical Analyses

Data were analyzed using GraphPad Prism version 8.0.1 (GraphPad Software; San Diego, CA, USA). The survival curves for each immunized group were compared to those of non-immunized mice in search of statistical differences using Log-rank test. Data from the Multiplex mouse cytokines and ICCS assays were analyzed using Mann–Whitney non-parametric test. Comparisons of mean responses between groups in the viremia analysis were performed using an independent-samples Student’s t test. A *p*-value lower than 0.05 was considered significant in all cases.

## 3. Results

### 3.1. Evaluation of BTV-4 NS1 and NS1-Nt Expression from Recombinant ChAdOx1 and MVAs

**Figure 2:**
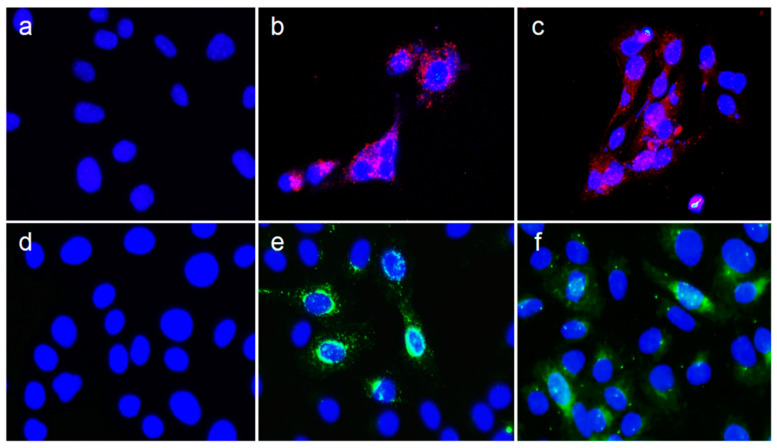
Analysis of BTV-4 NS1 and NS1-Nt protein expression by indirect immunofluorescence assay. Top Row: Detection of recombinant protein in DF-1 cells infected with (**a**) MVA-wt (negative control), (**b**) MVA-NS1 or (**c**) MVA-NS1-Nt at a MOI of 1. Bottom row: Detection of recombinant protein in HEK-293 cells infected with (**d**) ChAdOx1-wt (negative control), ChAdOx1-NS1 (**e**) or (**f**) ChAdOx1-NS1-Nt at a MOI of 1. Viral protein was detected using a hyperimmune serum against BTV-16; nucleus stained with DAPI; Alexa Fluor 594 goat conjugated anti-mouse IgG (red) was used to detect protein in DF-1 cells; Alexa Fluor 488 goat conjugated anti-mouse IgG (green) was used to detect protein in HEK-293 cells. Magnification 400×.

In order to evaluate the expression of NS1 and NS1-Nt of BTV-4 from ChAdOx1 and MVA vectors in infected HEK-293 and DF-1 cells, respectively, immunofluorescence assays were performed. The typical punctuated signal for NS1 and NS1-Nt was observed in HEK-293 cells infected with ChAdOx-NS1 (Figure 2b) and ChAdOx-NS1-Nt (Figure 2c) Expression of the NS1 and NS1-Nt BTV proteins was also observed in DF-1 cells infected with MVA-NS1 (Figure 2e) and MVA-NS1-Nt (Figure 2f). Cells infected with MVA wild type (wt) (Figure 2d) or non-infected (Figure 2a) did not show any specific fluorescence of NS1 or NS1-Nt. These data confirm the efficient expression of the BTV proteins cloned into the candidate vaccine vectors used for immunization of IFNAR(-/-) mice and sheep.

### 3.2. Immunization with ChAdOx1 or ChAdOx1/MVA Expressing Proteins NS1 or NS1-Nt of BTV-4 Protects IFNAR(-/-) Mice against Heterologous BTV-4M Infection

**Figure 3:**
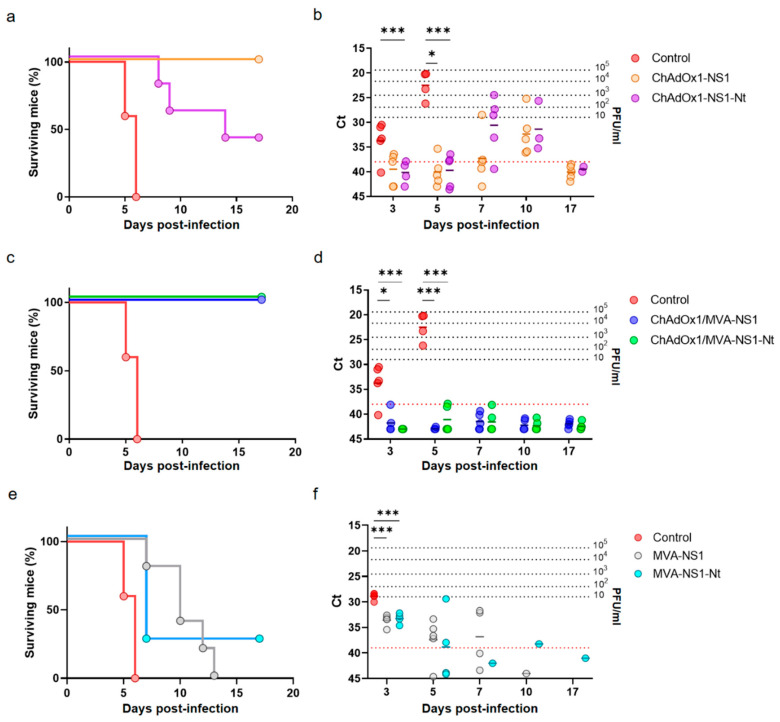
Protection of immunized IFNAR(-/-) mice against a lethal challenge with BTV-4M. Groups of 5 mice (*n* = 5) were immunized with (**a**,**b**) a single dose of 10^8^ IU per mouse of ChAdOx1-NS1 or ChAdOx1-NS1-Nt, (**c**,**d**) following a heterologous prime-boost regimen consisting of an initial dose of 10^8^ IU per mouse of ChAdOx1-NS1 or ChAdOx1-NS1-Nt (prime) followed by a second dose of 10^7^ PFU per mouse of MVA-NS1 or MVA-NS1-Nt (boost), respectively, administered 28 days apart, or (**e**,**f**) a single dose of 10^7^ PFU per mouse of MVA-NS1 or MVA-NS1-Nt. Immunized and non-immunized mice were challenged with a lethal dose (10 PFU) of BTV-4M as described in Materials and Methods. (**a**,**c**,**e**) Survival rates after infection. Statistical differences between the curves were calculated using Log-rank test. (**b**,**d**,**f**) Detection of BTV-4M in blood of non-immunized and immunized IFNAR(-/-) mice after challenge by RT-qPCR. Expression of mRNA of segment 5 (encoding NS1 protein) was quantified at 3, 5, 7, 10 and 17 d.p.i Results are expressed as Ct (left y-axis) and PFU equivalents (right *y*-axis and dotted horizontal lines). Cut-off Ct ≥ 38 (dotted pink line). Differences between groups were calculated by multiple t test analysis using the Sidak–Bonferroni method. * *p*-value < 0.033; *** *p*-value < 0.001.

Groups of adult IFNAR(-/-) mice were immunized with ChAdOx1-NS1 or ChAdOx1-NS1-Nt by intramuscular injection on the right leg. After four weeks, some groups of mice were boosted intraperitoneally with a dose of the corresponding MVA-NS1 or MVA-NS1-Nt, respectively. Four weeks after immunization (prime-only) and two weeks post-boost (prime-boost), both immunized and control IFNAR(-/-) mice were challenged subcutaneously with a lethal dose of BTV-4M. Non-immunized animals showed clinical signs including ruffled hair, lethargy, and eye swelling and all of them died by day 6 post-challenge. However, 100% of the animals immunized following the heterologous prime-boost strategy were completely protected against lethal challenge and none of them showed clinical signs. Mice immunized with a single dose of ChAdOx1-NS1 also overcome the infection, while immunization with a single dose of ChAdOx1-NS1-Nt led to a delay in appearance of clinical signs, but some mice began to show clinical signs by day 5 post-challenge and 60% of them succumbed to the infection after 14 days (Figure 3a). In addition, the presence of virus in the blood of mice after challenge with BTV-4M was analyzed by RT-qPCR. Non-immunized mice showed viremia at day three after infection and the titer of virus in blood increased thereafter until the death of the animals. A delay was observed in the onset of viremia in mice immunized with a single dose of ChAdOx1-NS1 or ChAdOx1-NS1-Nt, becoming viremic at day 7 and 10, respectively. Surviving mice returned to negativity at day 15 post infection (Figure 3b). In contrast, all animals immunized with the heterologous prime-boost ChAdOx1/MVA-NS1 or ChAdOx1/MVA-NS1-Nt yielded negative results at all analyzed days post-challenge (Ct ≥ 38) (Figure 3c,d). These data indicate that, despite just a single dose of ChAdOx1-NS1 is enough to confer protection against a lethal dose of BTV-4M, the heterologous prime-boost immunization with ChAdOx1/MVA-NS1 or ChAdOx1/MVA-NS1-Nt confers not only full protection against BTV-4M but also abrogates viremia and clinical signs. To confirm that the improvement in protection was due to an MVA booster effect over the ChAdOx1 priming, IFNAR (-/-) mice were immunized with a single dose of MVA-NS1 or MVA-NS1-Nt and two weeks after immunization animals were challenged with a lethal dose of BTV-4M. Although a delay in appearance of clinical signs and viremia was observed in the MVA immunized mice (Figure 3f), only 20% of the mice immunized with MVA-NS1-Nt and none of those immunized with MVA-NS1 survived to the infection with BTV-4M (Figure 3e). All this data indicates that while immunization with MVA-NS1 or MVA-NS1-Nt does not completely protect mice from infection with BTV-4M, it does improve the protection of animals with ChAdOx1-NS1 or ChAdOx1-NS1-Nt.

**Figure 4:**
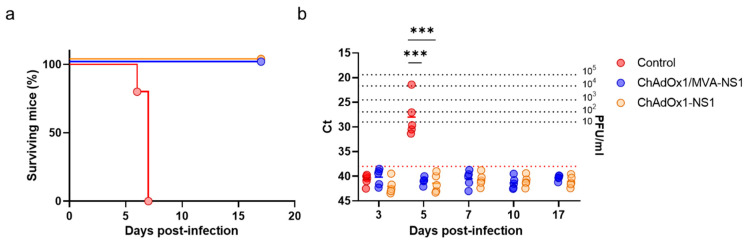
Protection of immunized IFNAR(-/-) mice against a lethal challenge with BTV-8. Groups of mice (*n* = 5) were immunized with a single dose of 10^8^ IU per mouse of ChAdOx1-NS1 or following a heterologous prime-boost regimen consisting of an initial dose of 10^8^ IU per mouse of ChAdOx1-NS1 (prime) followed by a second dose of 10^7^ PFU per mouse of MVA-NS1 (boost), administered 28 days apart. Immunized and non-immunized mice were challenged with a lethal dose (10 PFU) of BTV-8 at day 30 post-immunization (prime-only) or at day 15 post-boost (prime-boost). (**a**) Survival rates after infection. Statistical differences between the curves were calculated using Log-rank test. (**b**) Detection of BTV-4M in blood of non-immunized and immunized IFNAR(-/-) mice after challenge by RT-qPCR. Expression of mRNA of segment 5 (encoding NS1 protein) was quantified at 3, 5, 7, 10 and 17 d.p.i. Results are expressed as Ct (left y-axis) and PFU equivalents (right *y*-axis and dotted horizontal lines). Cut-off Ct ≥ 38 (dotted pink line). Differences between groups were calculated by multiple t test analysis using the Sidak–Bonferroni method. *** *p*-value < 0.001.

Consecutively, in order to confirm that NS1 delivered by the viral vectors ChAdOx1 or MVA induces full multiserotype protection against BTV, IFNAR(-/-) mice were immunized with ChAdOx1-NS1 or ChAdOx1/MVA-NS1 or non-immunized and challenged with a lethal dose of BTV-8. Non-immunized animals showed viremia at day five after infection and all of them died by day seven post-challenge. In contrast, all animals immunized with ChAdOx1-NS1 or the heterologous prime-boost ChAdOx1/MVA-NS1 survived to the infection with BTV-8 (Figure 4a) and viremia was not detected at any time analyzed post-challenge (Figure 4b). These results confirm that immunization of IFNAR(-/-) mice with ChAdOx1-NS1 or ChAdOx1/MVA-NS1 confers protection against multiple serotypes of BTV.

### 3.3. Heterologous Prime-Boost Vaccination with ChAdOx1/MVA Expressing NS1 or NS1-Nt Induces Strong Cellular Response against NS1

We have previously determined the importance of the non-structural protein NS1 in CD8+ T cell-mediated protection against multiple BTV serotypes [14]. In order to analyze the protective immune responses induced in mice immunized with ChAdOx1 or ChAdOx1/MVA expressing NS1 or NS1-Nt, we performed virus neutralization tests (VNT), ELISA assay to detect antibodies specific of NS1, and intracellular cytokine staining assays (ICCS). The presence of specific antibodies against NS1 in serum was analyzed 14 days post-boost or 30 days post-immunization (single dose) by ELISA. Antibody levels were significantly higher (*p* = 0.029; mean O.D. = 0.665) in mice immunized with ChAdOx1/MVA-NS1 compared to control mice (mean O.D. = 0.211), and the increase in the levels of antibodies was also close to significant (*p* = 0.057; mean O.D. = 0.428) after immunization with ChAdOx1/MVA-NS1-Nt when compared with non-immunized mice. However, no significant differences in the levels of antibodies against NS1 were observed between the sera of mice immunized with a single dose of ChAdOx1-NS1 (*p* = 0.486; mean O.D.= 0.186) or ChAdOx1-NS1-Nt (*p* = 0.114; mean O.D. = 0.275) and those of control mice. Although both prime/boost strategies induced antibodies against NS1, these antibodies did not show neutralizing activity against BTV-4M as determined by a VNT assay. (Supplementary Materials Figure S3).

**Figure 5:**
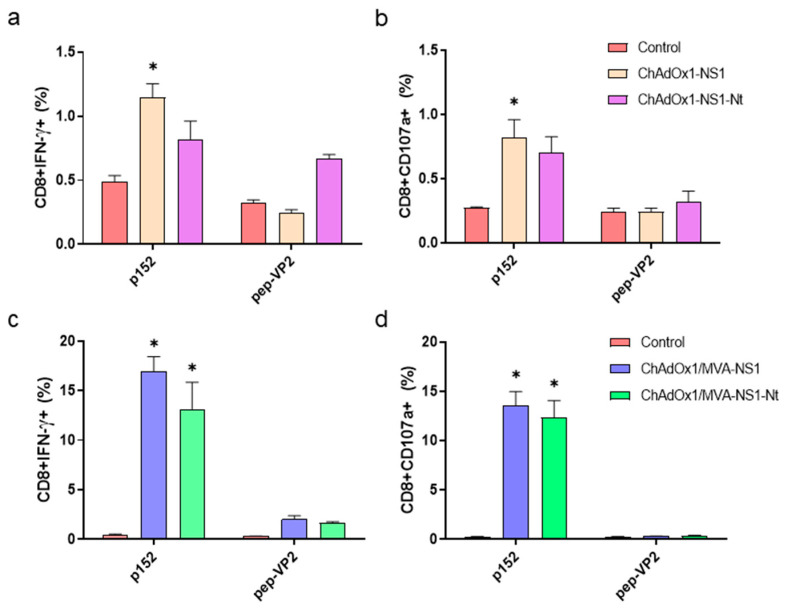
Cellular immune responses against BTV in immunized mice. IFNAR(-/-) mice were non-immunized or immunized with a single dose of ChAdOx1-NS1 or ChAdOx1-NS1-Nt (**a**,**b**), or following a heterologous prime-boost of ChAdOx1/MVA-NS1 or ChAdOx1/MVA-NS1-Nt (**c**,**d**). Four weeks post-immunization (prime-only) or two weeks after boosting with the appropriate MVA (prime-boost), spleens were harvested and splenocytes were stimulated with 10 μg/mL of p152 (immunodominant peptide from NS1 protein), with 10 μg/mL of a non-relevant peptide from protein VP2 (pep-VP2) protein, or left untreated in RPMI medium. Intracellular staining of IFN-γ (**a**,**c**) or CD107a (**b**,**d**), in CD8+ T cells was analyzed by flow-cytometry. Graphs show the percentage of CD8+IFN-γ+ or CD8+ CD107a+ lymphocytes. Bars represent the mean values of each group and error bars represent standard error of the mean (SEM). Asterisks denote significant differences between stimulated splenocytes of immunized and control mice (*p* < 0.05) (The Mann–Whitney U test). Gate was set making use of double-negative splenocytes corresponding to non-immunized mice exposed to RPMI medium. * *p*-value < 0.05.

To determine the ability of the candidate vaccines to elicit specific T cell responses, splenocytes of immunized and non-immunized mice were re-stimulated with the NS1 immunodominant peptide p152 (9-mer peptide GQIVNPTFI) or with a non-relevant peptide from protein VP2 of BTV-4 [14] and we determined IFN-γ production as well as CD107a cytotoxic marker expression in CD8+ T cells by ICCS. The peptide p152 elicited a low but significant response in CD8+ T cells of ChAdOx1-NS1 but not in ChAdOx1-NS1-Nt immunized mice in comparison with control animals (Figure 5). These could explain the full protection against BTV-4M observed only in the ChAdOx1-NS1 immunized mice. In contrast, we observed a strong induction of CD8+IFNγ+ and CD8+CD107a+ cells upon re-stimulation of ChAdOx1/MVA-NS1 and ChAdOx1/MVA-NS1-Nt immunized mouse splenocytes with p152. The re-stimulation of splenocytes from control mice with p152 or from immunized mice with the irrelevant peptide VP2 showed negligible responses to the peptides. These data show that the heterologous prime-boost immunization with ChAdOx1/MVA-NS1 or ChAdOx1/MVA-NS1-Nt elicits a strong cytotoxic CD8+ T cell response in mice that completely protects against a lethal challenge with BTV-4M in absence of neutralizing antibodies.

**Figure 6:**
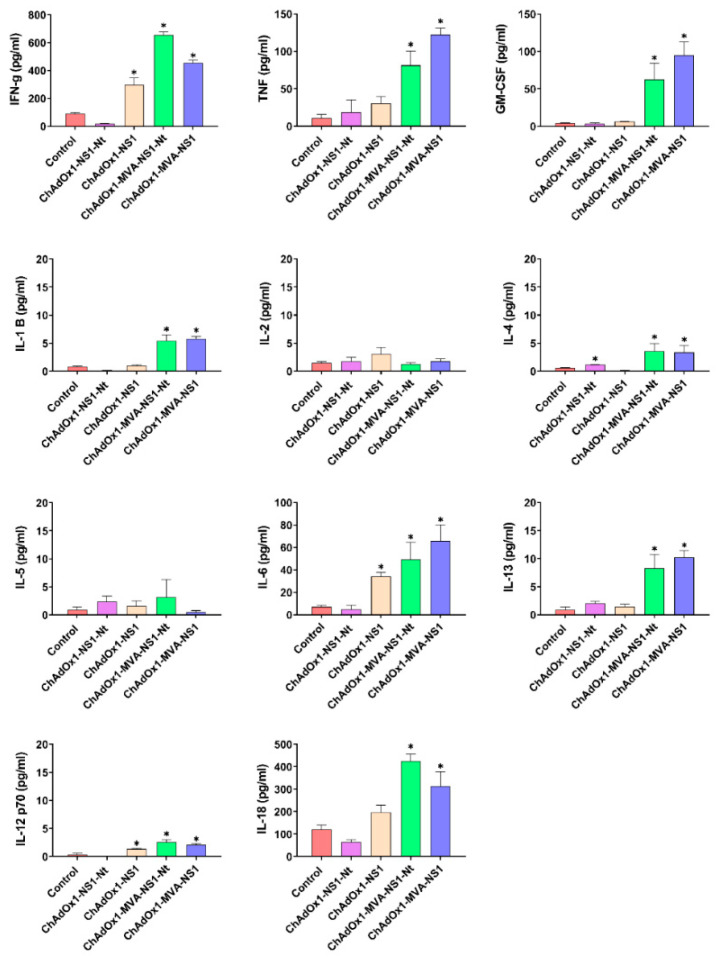
Cytokine production measured in supernatants from mice splenocytes two weeks post-immunization (prime-only) or after boosting with MVAs. Prior to quantification, splenocytes from immunized and non-immunized mice were stimulated for 72 h with 10 μg/mL of NS1-152 peptide or left untreated. Values of non-stimulated samples were subtracted from values of stimulated samples. Bars represent mean values of each group and error bars represent SEM. Asterisks denote significant differences between stimulated splenocytes of immunized and non-immunized control mice (* *p* < 0.05) (The Mann–Whitney U test).

To further characterize the cellular immune response, splenocytes of immunized and non-immunized mice were cultured and re-stimulated with p152 for 72 h and the supernatants were analyzed for the presence of cytokines (Figure 6). After p152 stimulation, splenocytes from ChAdOx1/MVA-NS1 or ChAdOx1/MVA-NS1-Nt secreted high levels of IFN-γ, TNF, GM-GSF, IL-6 and IL-18, and moderate but significant levels of IL-1β, IL-12, IL-13 and IL-4. These results suggest that the assayed prime-boost immunization directs T cell responses towards the phenotype Th1. Splenocytes from ChAdOx1-NS1-Nt immunized mice did not secrete significant levels of these cytokines after stimulation, in contrast with those from ChAdOx1-NS1 immunized animals that secreted significant levels of IFN-γ, IL-6 and IL-12. The difference found in the secretion of cytokines after stimulation between ChAdOx1-NS1 and ChAdOx1-NS1-Nt immunized animals could explain the stronger protection of ChAdOx1-NS1 against BTV-4M infection.

### 3.4. Immunization with ChAdOx1 or ChAdOx1/MVA Expressing Proteins NS1 or NS1-Nt of BTV-4 Elicit Long-Lasting Protection in IFNAR(-/-) Mice against Heterologous BTV-4M Infection

**Figure 7:**
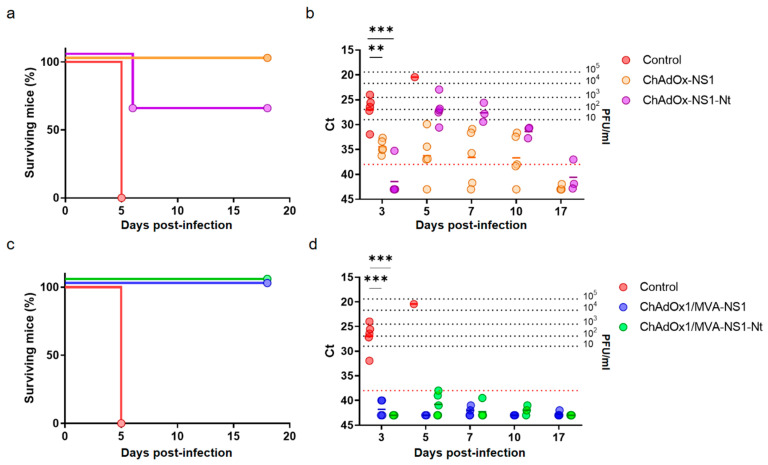
Long-lasting protection of immunized IFNAR(-/-) mice against a lethal challenge with BTV-4M. Groups of IFNAR(-/-) mice (*n* = 5) were immunized with (**a**,**b**) a single dose of 10^8^ IU per mouse of ChAdOx1-NS1 or ChAdOx1-NS1-Nt or (**c**,**d**) following a heterologous prime-boost regimen consisting of an initial dose of 10^8^ IU per mouse of ChAdOx1-NS1 or ChAdOx1-NS1-Nt (prime) followed by a second dose of 10^7^ PFU per mouse of MVA-NS1 or MVA-NS1-Nt (boost), respectively, administered 28 days apart. Immunized and non-immunized mice were challenged with a lethal dose (10 PFU) of BTV-4M at day 160 post-immunization. (**a**,**c**) Survival rates after infection. Statistical differences between the curves were calculated using Log-rank test. (**b**,**d**) Detection of BTV-4M in blood of non-immunized and immunized IFNAR(-/-) mice after challenge by RT-qPCR. Expression of mRNA of segment 5 (encoding NS1 protein) was quantified at 3, 5, 7, 10 and 17 d.p.i. Results are expressed as Ct (left *y*-axis) and PFU equivalents (right y-axis and dotted horizontal lines). Cut-off Ct ≥ 38 (dotted pink line). Differences between groups were calculated by multiple t test analysis using the Sidak–Bonferroni method. ** *p*-value < 0.02; *** *p*-value < 0.001.

The partial or full protection observed in the immunized animals challenged four (prime only) or two weeks (prime-boost) after the last immunization encouraged us to analyze whether the same immunization could provide long-lasting protection against BTV-4M infection. Four months after the last immunization, mice immunized with ChAdOx1 or ChAdOx1/MVA expressing NS1 or NS1-Nt were challenged with a lethal dose of BTV-4M. Survival rates and viremia were similar to those observed previously when the immunized mice were infected four weeks after immunization and two weeks after booster (Figure 7a,b). All control mice presented viremia and clinical signs at 3 d.p.i., presenting ruffled hair coat, lethargy and eye swelling, and 100% of these mice died at day 5 post-challenge. A single dose of ChAdOx1-NS1-Nt protected 60% of the animals but immunized animals showed viremia at day 5 post-infection. Animals immunized with a single dose of ChAdOx1-NS1 survived to the infection with BTV-4M. Although 80% of these mice showed low levels of viremia at 3 d.p.i., below 10 PFU/mL, they returned to negativity for the next days of the experiment. Most importantly, all animals immunized with the heterologous prime-boost ChAdOx1/MVA-NS1 or ChAdOx1/MVA-NS1-Nt were protected against BTV-4M and they yielded negative results of viremia at all analyzed days post-challenge (Ct ≥ 38) (Figure 7c,d).

### 3.5. ChAdOx1/MVA-NS1 and ChAdOx1/MVA-NS1-Nt Confers Protection in Sheep after BTV-4M Challenge

**Figure 8:**
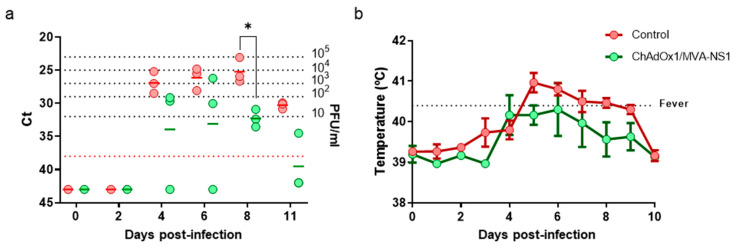
Analysis of protection elicited by ChAdOx1/MVA-NS1 immunization in sheep after infection with BTV-4M. (**a**) Viremia analyzed by RT-qPCR. Expression of mRNA of segment 5 (encoding NS1 protein) was quantified at 0, 2, 4, 6, 8 and 11 d.p.i. with BTV-4M. Results are expressed as Ct (left *y*-axis) and PFU equivalents (right *y*-axis and dotted horizontal lines). Cut-off Ct ≥ 38 (dotted pink line). Differences between groups were calculated by multiple t test analysis sing the Sidak–Bonferroni method. * *p*-value < 0.033 (**b**) Mean rectal temperatures responses in ChAdOx1/MVA-NS1 vaccinated and non-vaccinated sheep from 0 to 10 d.p.i. with BTV-4M. The fever threshold (dotted black line) was set to ≥40.50 °C based on the mean plus three standard deviations of the rectal temperatures recorded in the six sheep for one week before challenge. Dots indicate media of the group. Error bars represent SEM. Differences between groups were calculated by multiple t test analysis using the Sidak–Bonferroni method.

The protection conferred on mice by ChAdOx1/MVA-NS1 and ChAdOx1/MVA-NS1-Nt against BTV-4M led us to analyze their efficacy as a potential vaccine for ruminants. Although we did not observe differences in protection between ChAdOx1/MVA-NS1 and ChAdOx1/MVA-NS1-Nt, the level of protection observed with a single dose of ChAdOx1 was higher in mice immunized with ChAdOx1-NS1. For this reason, we chose the heterologous prime-boost with ChAdOx1/MVA-NS1 as the best immunization strategy to be analyzed in sheep. Three sheep were inoculated with a prime of ChAdOx1-NS1 at day 0 and a boost of MVA-NS1 four weeks apart and neither clinical display nor adverse effects were noticed in any animal. Five weeks after second vaccination, animals were challenged with 10^5^ PFU of BTV-4M and viremia and rectal temperature were analyzed for two weeks. At 5 d.p.i., all non-vaccinated sheep developed pyrexia with a mean value of 40.96 °C and fever persisted up to 8 d.p.i. Vaccinated sheep showed lower temperature and two out of three vaccinated sheep did not show pyrexia with temperatures below the baseline levels throughout the experiment (fever threshold ≥40.4 °C, described in materials and methods) (Figure 8b). Viremia was tested by real-time RT-qPCR and viral RNA was detected in non-vaccinated sheep from 4 d.p.i., reaching levels between 10^3^ and 10^4^ PFU per ml of blood between days 4 to 8 post-challenge (Figure 8a). Lower levels of viral RNA were detected in ChAdOx1/MVA-NS1 immunized group (mean below 10 PFU/mL) compared to control sheep, indicating that the vaccinated sheep were protected against BTV-4M. These results indicate that immunization of sheep with a heterologous prime-boost of ChAdOx1/MVA expressing NS1 confers protection against BTV-4M and reduces viremia and clinical signs.

## 4. Discussion

One of the key drawbacks in generating an effective universal vaccine for BTV is the large number of serotypes. Currently, no BTV universal vaccine is available. Ideally, such a vaccine would protect against a wide variety of different BTV serotypes. The marketed vaccines, LAVs and inactivated vaccines, induce strong immune responses against immunogenic BTV proteins, mainly VP2. Therefore, their protective efficacy is essentially based on a strong induction of neutralizing antibodies, being serotype-specific. We have tried to address this serotype specificity of the classical vaccines through the design and generation of T-cell vaccines using ChAdOx1 and MVA vectors encoding protein NS1, or its truncated version NS1-Nt, which are highly conserved among viral serotypes.

The generation of functional and long-lasting CD8+ T cells is necessary to ensure an adequate protection against intracellular pathogen as they constitute an important component of the adaptive immune response, thus contributing to the clearance of viruses [45,46]. Particularly, the combination of both virus neutralizing antibodies and cytotoxic T lymphocytes (CTLs) is crucial for the development of a long lasting immunity in animals infected with BTV [47,48]. We have previously demonstrated the importance of the non-structural protein NS1 in CD8+ T cell-mediated protection against multiple BTV serotypes when vectorized as a recombinant MVA vaccine [14]. Immunization of mice following a homologous prime-boost with MVA-NS1 or MVA-NS1-Nt conferred protection against multiple BTV serotypes and reduced or abrogated both viremia and clinical signs. In the current study we show that although a single dose of ChAdOx1 expressing the NS1-Nt protein of BTV-4 provided only partial protection against BTV-4M, whose segment 5 comes from BTV-1, the immunization with ChAdOx1 expressing NS1 protein fully protected IFNAR(-/-) mice against a lethal dose of BTV-4M. Furthermore, this degree of protection was maintained for at least four months after immunization, which indicates that protection was durable. Although we did not observe sterile protection against BTV-4M in ChAdOx1-NS1 immunized mice, the virus titers detected in blood were minimum (below 10 PFU/mL) and none of the immunized animals displayed signs of disease. The efficacy of a single dose of ChAdOx1 in protection has been demonstrated for a wide range of viruses such as hepatitis C virus [49], influenza A virus [28], ebola virus [50], HIV [51], Chikungunya [29], Rift Valley fever virus and MERS–CoV [32], among others. In the case of many of these vaccines, the protection derived from the immunization was due to the induction of neutralizing antibodies against the virus and not only to an induction of a cellular immune response as is the case of ChAdOx1-NS1 where neutralizing activity was not detected in sera of immunized animals.

Although a single immunization with ChAdOx1-NS1 might be sufficient for protection against BTV-4M, multiple studies using the ChAdOx1/MVA prime-boost regimen have shown to be superior compared to a singular vector prime. MVA given as a boost following a ChAdOx1 immunization elicited strong cell-mediated immune responses and mice were fully protected against the infection with a lethal dose of BTV-4M. The booster with MVA expressing NS1 or NS1-Nt in animals previously primed with ChAdOx1-NS1 or ChAdOx1-NS1-Nt significantly induced a cellular immunity, particularly IFN-γ–producing CD8+ T cells. Furthermore, cytokine secretion after stimulation of splenocytes with the peptide 152, an immunodominant CD8+ T cell epitope from NS1, was higher in animals boosted with MVA than in those immunized with a single dose of ChAdOx1-NS1 or ChAdOx1-NS1-Nt. After p152 stimulation, splenocytes from ChAdOx1/MVA-NS1 or ChAdOx1/MVA-NS1-Nt secreted high levels of IFN-γ, TNF-α, GM-CSF, IL-6 and IL-18, which indicates an induction of a polyfunctional CD8+ T cell response. Our data suggests that T cell responses in IFNAR(-/-) mice vaccinated with ChAdOx1 and MVA viral vectors induce robust cellular Th1 immune responses. This is in agreement with previous works supporting the augmentation of T cell-mediated responses by MVA after a ChAdOx1 prime, in heterologous prime-boost strategies in both mice and human [52,53,54,55,56,57,58,59].

The importance of the non-structural protein NS1 in CD8+ T cell-mediated protection against BTV has been previously showed [14,18,19]. Additionally, the NS1 protein is highly conserved among different BTV serotypes and so are the epitopes associated with T-cell responses [14,19,44,60]. Animals immunized with ChAdOx1-NS1 or ChAdOx1/MVA-NS1 were not only protected against infection with BTV-4(M) but also against a lethal dose of BTV-8. Protection was even stronger against BTV-8 with no viremia or clinical signs observed in mice immunized with a dose of ChAdOx1-NS1 or a prime boost with ChAdOx1/MVA-NS1 after challenge with BTV-8. For this reason, the NS1 protein can be proposed as a candidate antigen in the development of universal vaccines against BTV.

Since the protection against BTV induced by this prime-boost ChAdOx1/MVA-NS1 was more effective than just a single dose with ChAdOx1-NS1, we decided to test the protection elicited by ChAdOx1/MVA-NS1 against BTV-4M in sheep, one of the BTV natural hosts. Both viral vectors ChAdOx1 and MVA have been used previously in ruminants as vaccines. It has been reported that a single-dose immunization with ChAdOx1 vaccine encoding RVFV envelope glycoproteins elicited high titers of RVFV-neutralizing antibodies and provided protection in sheep, goats and cattle [35]. Moreover, MVA expressing Gn and Gc RVFV glycoproteins administered as a boost following a DNA vaccination was able to reduce clinical signs and viremia in adult sheep challenged long after immunization [61]. In this work, the heterologous prime-boost immunization with ChAdOx1/MVA-NS1 was successful in providing immunity against BTV in sheep. Vaccinated animals clearly showed lower viremia and clinical signs in comparison with non-vaccinated animals after challenge with BTV-4M. Although viremia was not fully abrogated in the immunized sheep, the titers of virus found was 10^3^ PFU/mL or lower. This reduction of viremia not only prevents the development of clinical signs in the immunized animals but may also reduce viral acquisition by *Culicoides* bites. Experimental infections of *Culicoides sonorensis* with BTV-11 and BTV-1 infected blood showed that the efficiency of infection of midges was dose-dependent and the 50% Midge Alimentary Infective Dose (MAID50) was estimated to a blood meal titer around 2 × 10^5^ TCID50/mL [62,63]. In the case of BTV-4M, a virus titer between 10^5^ and 10^6^ TCID50 is enough to ensure BTV infection rates of 100% for *C. imicola* and ~83% for *C. obsoletus*/*scoticus* [64]. Consistent with these results, the detected viremia in ChAdOx1/MVA-NS1 vaccinated sheep was much lower than the minimal dose required for the infection of *Culicoides*, which entails an unlikely transmission of BTV.

An ideal BTV vaccine, besides its proven effectiveness and safety, is bound to be multiserotype and should allow to distinguish between infected and vaccinated animals (DIVA strategy). Indeed, the combination of serologic tests based on the recognition of antibodies specific of NS1 [40] and the commercially available ELISA diagnostic tests targeting antibodies specific of VP7 could facilitate this DIVA strategy. Regarding efficacy and safety, ChAdOx1 and MVA have been widely used as vaccine vectors with provided assurance and immunogenicity. In this sense, ChAdOx1 safety has been demonstrated even in pregnant animals as long as vaccination of pregnant sheep and goats with ChAdOx1 expressing RVFV glycoproteins provided protection against RVFV and avoided fetal loss, data that support the ongoing development of ChAdOx1 vaccines for use not only in humans but also in livestock [65]. In this work, after immunization with ChAdOx1/MVA-NS1 neither clinical display nor adverse effects were noticed in any animal, thus confirming the safety of these vaccine vectors. The dose of the viral vectors ChAdOx1 (10^9^ UI/sheep) and MVA (10^8^ PFU/sheep) used in our immunization strategy have been previously assessed in other sheep immunization assays and have been shown to be safe and efficient against RVFV [61,65]. These doses are below those used in human studies where even doses of 10^11^ viral particles have been used for vaccines based on ChAdOx1 [66]. The augmented antigenic load has been suggested to affect qualitative aspects of CD8+ T-cell memory/effector responses [67,68] and, despite our results indicate that sheep immunized with ChAdOx1/MVA-NS1 were protected against BTV-4M infection, it would be interesting to analyze whether higher doses of vaccine are still safe for ruminants and completely reduce viremia after BTV infection.

Several experimental BTV vaccines are under development in order to improve the marketed vaccines and overcome their disadvantages. Among the different types of vaccines under development, those based on viral vectors expressing one or more BTV proteins and vaccines based on attenuation of BTV by reverse genetics (e.g., disabled infectious single cycle (DISC) and disabled infectious single animal (DISA) vaccines) [15,69,70] stand out. These vaccines are effective, safe, make possible a DIVA strategy, but they do not elicit multiserotype protection, even though application of DISC and DISA vaccines for multiple serotypes have been successfully studied in ruminants. Although further studies on this vaccination strategy regarding vaccination dose and the assessment of whether it induces long lasting protection in the natural host will be necessary, the data showed in this work demonstrate that the development of vaccines based on protein NS1 have a high potential to induce a strong CD8+ T cell-mediated responses against BTV, so that they could accomplish maximal cross-protective efficacy. What is more, we demonstrate that the utilization of ChAdOx1 and MVA vaccine vectors, widely used in the development of human vaccines, are effective and safe candidates for the generation of vaccines against livestock diseases such as BTV.

## 5. Conclusions

Several experimental BTV vaccines are under development in order to improve the marketed vaccines and overcome their disadvantages. Among the different types of vaccines under development, those based on viral vectors expressing one or more BTV proteins and vaccines based on attenuation of BTV by reverse genetics (e.g., disabled infectious single cycle (DISC) and disabled infectious single animal (DISA) vaccines) [15,69,70] stand out. These vaccines are effective, safe, make possible a DIVA strategy, but they do not elicit multiserotype protection, even though application of DISC and DISA vaccines for multiple serotypes have been successfully studied in ruminants. Although further studies on this vaccination strategy regarding vaccination dose and the assessment of whether it induces long lasting protection in the natural host will be necessary, the data showed in this work demonstrate that the development of vaccines based on protein NS1 have a high potential to induce a strong CD8+ T cell-mediated responses against BTV, so that they could accomplish maximal cross-protective efficacy. What is more, we demonstrate that the utilization of ChAdOx1 and MVA vaccine vectors, widely used in the development of human vaccines, are effective and safe candidates for the generation of vaccines against livestock diseases such as BTV.

## Supplementary Materials

The following are available online at https://www.mdpi.com/2076-393X/8/3/346/s1, Figure S1: Immunization schedules and groups of mouse studies, Figure S2: Immunization schedule and groups of sheep studies, Figure S3: Humoral responses against BTV-4M elicited by MVA and ChAdOx1 in IFNAR(-/-) mice.
