# Supplementary material for: Heterologous Combination of ChAdOx1 and MVA Vectors Expressing Protein NS1 as Vaccination Strategy to Induce Durable and Cross-Protective CD8+ T Cell Immunity to Bluetongue Virus
Source: Vaccines (Basel). 2020 Jun 29;8(3):346. doi: 10.3390/vaccines8030346 (PMC7564706; doi:10.3390/vaccines8030346)
Supplement: Supplementary file 1 [file vaccines-08-00346-s001.pdf]

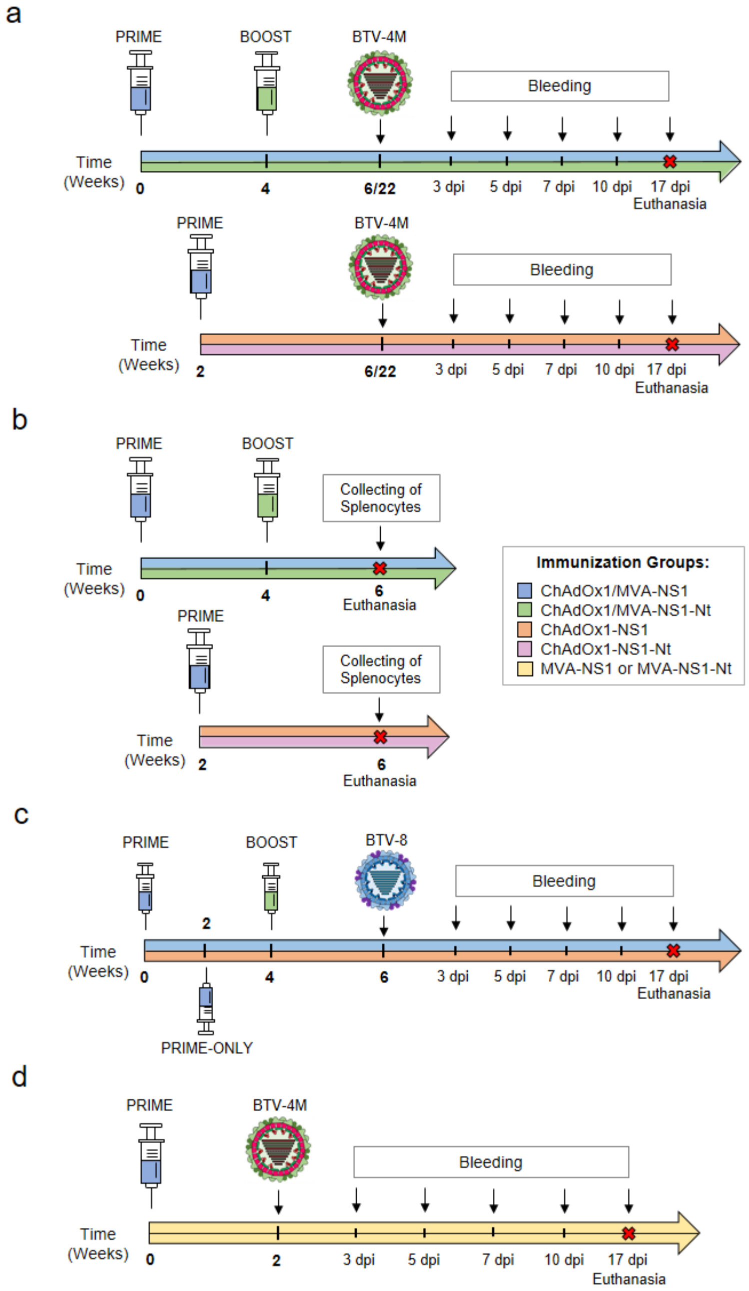

Supplementary Figure S1. Immunization schedules and groups of mouse studies. (a) Four groups of IFNAR(-/-) mice (n=5) were immunized with a single dose of ChAdOx1-NS1 or ChAdOx1-NS1-Nt or with a heterologous prime-boost of ChAdOx1/MVA-NS1 or ChAdOx1/MVA-NS1-Nt and challenged with a lethal dose (10 PFU) of BTV-4M four weeks after prime (prime-only) or two weeks after booster (prime-boost). A group of mice was non-immunized (control). The bleedings were carried out on the different indicated post-infection days. (b) Four groups of IFNAR(-/-) mice (n=4) were immunized as indicated above and splenocyte collection was performed four weeks after prime (prime-only) or two weeks after booster (prime-boost) for the cellular immune response assays. A group of mice was non-immunized (control). (c) Two groups of IFNAR(-/-) mice (n=5) were immunized with a single dose of ChAdOx1-NS1 or following a heterologous prime-boost of ChAdOx1/MVA-NS1 and challenged with a lethal dose (10 PFU) of BTV-8 four weeks after prime (prime-only) or two weeks after booster (prime-boost). A group of mice was non-immunized (control). The bleedings were carried out on the different indicated post-infection days. (d) Two groups of IFNAR(-/-) mice (n=5) received a single dose of MVA-NS1 or MVA-NS1-Nt. Challenge with a lethal dose (10 PFU) of BTV-4M was performed two weeks after immunization. A group of mice

was non-immunized (control). The bleedings were carried out on the different indicated post-infection days.

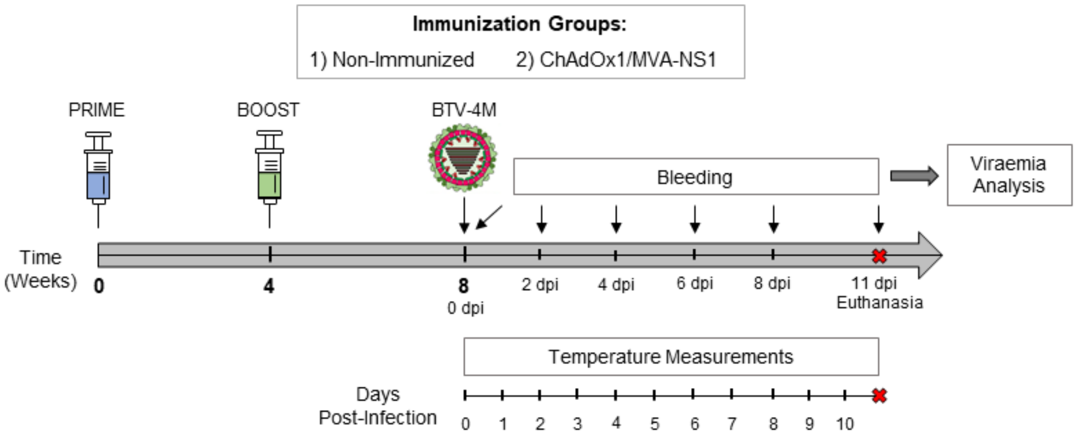

Supplementary Figure S2. Immunization schedule and groups of sheep studies. Two groups of sheep (n=3) were immunized following a heterologous prime-boost of ChAdOx1/MVA-NS1 or were left untreated (non-immunized). Immunization and challenge with BTV-4M were performed at the indicated time points in weeks. The bleedings were carried out on the different indicated post-infection days. Rectal temperature measurements were conducted at the indicated time points after infection.

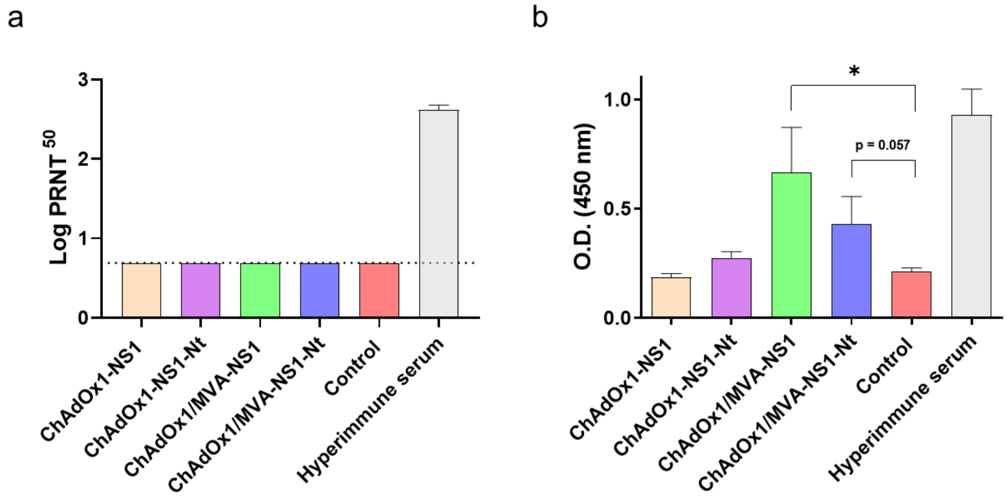

Supplementary Figure S3. Humoral responses against BTV-4M elicited by MVA and ChAdOx1 in IFNAR(-/-) mice. (a) Induction of virus neutralizing antibodies against BTV in animals immunized with a single dose of ChAdOx1-NS1 or ChAdOx1-NS1-Nt, or following a heterologous prime-boost of ChAdOx1/MVA-NS1 or ChAdOx1/MVA-NS1-Nt by plaque reduction neutralization assay. The columns represent the mean of the group and error bars that indicate the SEM. Cut-off: 0.69 ($\log_{10} 5$). (b) Induction of IgG NS1 antibodies by indirect ELISA in vaccinated animals. Sera dilution 1:50. Bars represent mean values of each group and SEM are shown as error bars. Asterisks denote statistical differences ($p < 0.05$) between the means of each group and the mean value of non-immunized mice, as calculated by Mann-Whitney non-parametric test. Control: non-immunized mice. Hyperimmune serum: serum from mice infected with BTV-4M.
